# The development of multiple drug use among anabolic-androgenic steroid users: six subjective case reports

**DOI:** 10.1186/1747-597X-3-24

**Published:** 2008-11-28

**Authors:** Kurt Skårberg, Fred Nyberg, Ingemar Engström

**Affiliations:** 1 School of Health and Medical Sciences, Psychiatric Research Centre, Örebro University, Örebro, Sweden; 2 Addiction Centre, Örebro County Council, Örebro, Sweden; 3 Department of Pharmaceutical Biosciences, Uppsala University, Uppsala, Sweden

## Abstract

**Background:**

The inappropriate use of anabolic androgenic steroids (AAS) was originally a problem among athletes but AAS are now often used in nonsport situations and by patients attending regular addiction clinics. The aim of this study was to improve understanding of the development of multiple drug use in patients seeking treatment at an addiction clinic for AAS-related problems.

**Methods:**

We interviewed six patients (four men and two women) with experience of AAS use who were attending an addiction clinic for what they believed were AAS-related problems. The patients were interviewed in-depth about their life stories, with special emphasis on social background, substance use, the development of total drug use and subjective experienced psychological and physical side effects.

**Results:**

There was significant variation in the development of drug use in relation to social background, onset of drug use, relationship to AAS use and experience of AAS effects. All patients had initially experienced positive effects from AAS but, over time, the negative experiences had outweighed the positive effects. All patients were dedicated to excess training and took AAS in combination with gym training, indicating that the use of these drugs is closely related to this form of training. Use of multiple drugs was common either in parallel with AAS use or serially.

**Conclusion:**

The study shows the importance of understanding how AAS use can develop either with or without the concomitant use of other drugs of abuse. The use of AAS can, however, progress to the use of other drugs. The study also indicates the importance of obtaining accurate, comprehensive information about the development of AAS use in designing treatment programmes and prevention strategies in this area.

## Background

Anabolic androgenic steroids (AAS) are synthetic derivatives of the male endogenous sex hormone testosterone, which exhibits both anabolic (protein-synthesizing) and androgenic (masculinizing) effects. These drugs were originally used only in the context of elite sports [1]. Today, however, AAS are used by a far wider range of groups outside of sports and athletics [2,3]. The use of AAS has therefore become a subject of considerable scientific interest in addiction and psychiatric research.

Epidemiological studies on this topic are notoriously difficult to conduct in a reliable manner since AAS usage is largely clandestine, partly because the drugs are illegal and partly because usage tends to take place in closed sub-cultural settings. Despite these problems, we know that the current use of AAS is relatively widespread in many countries, including Sweden, which is the site of the current study. AAS are found in both cities and smaller communities [3]. The majority of users are male [4,5] and most of the users begin using the drugs in their teens or early twenties [6].

It has been noted that AAS are often combined with alcohol [7] and other drugs of abuse [8] as well as with testosterone releasers, anti-estrogens [2,9] and other medications [10,11]. The reasons usually given for this are that the combination both increases the effects of AAS and decreases various physical and psychological side effects. Side effects from AAS use reported in men include impotence and infertility due to inhibited endogenous testosterone production. In women, increased virility, including deepening of the voice, changes in libido and clitoral enlargement, occurs as a consequence of AAS use [12]. Other known side effects include atherosclerosis, hypertension, dilated cardiomyopathy and sudden death. Psychiatric side effects include irritability, aggressiveness, mood swings, decreased impulse control and suicidal or homicidal behaviour [12].

AAS continue to be used, despite knowledge of these potentially serious side effects. The most commonly reported motives for using AAS are enhanced performance in sports, improved physical appearance, increased body size and aggressiveness, strengthened libido and an enhanced sense of well-being [3,13-15]. Other justifications for continuing with AAS include self-fulfilment accounts, condemnation of condemners (a way of shifting focus from the user's own deviant acts) and denial of injury [16].

Most studies regarding motives for using AAS and combinations of drugs are based on athletes. To the best of our knowledge, there are no studies exploring why and how non-athletic users have started to use a combination of drugs. It can therefore be said that the pathways for the development of mixed abuse are inadequately described. It has been proposed that AAS abuse can be a gateway to the use of other drugs of abuse [17-19] and alcohol abuse [20] but the empirical grounds for these conclusions are fairly weak.

Although AAS have been studied extensively in recent years, the perspectives of the users themselves are only sparsely described, despite in-depth knowledge among users about the drugs, their effects, their possible side effects and how they can be combined [6,8,21]. It has also been reported that AAS users find it problematic that doctors and other healthcare staff have a limited understanding of the issue [15,22-24]. There are thus many reasons to pay greater attention to the users' own stories about the development of their abuse patterns and how their use of different combinations of drugs has developed.

There are a few articles in the scientific literature based on case reports [16,25-27] describing the development of AAS use from the user's perspective. Todd [25] performed in-depth interviews with American weight-lifters concerning AAS use from the athletes' perspective. His conclusion was that the largest group of AAS users seems to be "average guys who just want to get bigger and stronger as fast as they can". Monaghan [16] interviewed 67 bodybuilders and weight-lifters concerning their motivation for use of AAS. One important finding was that most AAS users generally expressed a positive view about the effects of AAS. Olrich & Ewing [27] interviewed ten men about their experiences with these drugs. Nearly all of them described predominantly positive experiences. Their feelings of affirmation extended well beyond the walls of the gym and their narratives suggested feelings of elevated status in most social environments. The authors suggest that the users enjoyed benefits linked with the "embodiment of masculinity" in our culture. The authors therefore stress that all measures to address AAS abuse, both prevention and treatment, must be designed on the basis of these results, since the decision to stop using AAS means foregoing experiences of powerfully enhanced masculinity.

Grogan et al. [26] interviewed five women and six men using snowball sampling. Again, a major finding was that most of the users in this study reported largely positive experiences of AAS. The majority felt that moderate use of AAS was nonproblematic and that the risk of serious side effects was not a sufficient deterrent to put them off using the drugs. Information from the healthcare sector regarding AAS was generally disbelieved, particularly since it differed from their personal experience. The importance of noting users' largely positive experiences is stressed, and cooperation with the body building community was reported as being decisive for the outcome of any programme.

In earlier studies, we have described a group of AAS users from an addiction clinic in terms of their social backgrounds, current social situation [28] and total drug use pattern [29]. These studies revealed that AAS abusers often come from problematic family backgrounds, have a history of major problems in school, have considerable social problems in daily life and have common histories of polysubstance drug use. In the present study, we aim to complement these data by using in-depth interviews to focus on the users' own perspectives of their experiences with AAS.

The aim of the study was thus to let AAS users' own stories serve as a point of departure for examining the various consequences of development of drug usage among a group of people seeking help at an addiction clinic. The participants were selected to capture as wide a variation as possible in experiences.

## Methods

### Sample

This study is based on a total of 36 AAS-users, 34 men and 2 women, who were consecutively included from a psychiatric addiction clinic in Orebro county, central Sweden, a county of 275,000 inhabitants. All patients were attending the addiction clinic to seek help for what they believed to be AAS-related side effects. The inclusion criteria for participants were that they must: a) be over 16 years of age, b) be fluent in Swedish, c) have been using non-prescribed AAS within the last four months, alone or in combination with other doping agents, d) have been using AAS for at least four months and e) be under the care of the addiction clinic where a decision to commence treatment for their AAS use had been agreed upon following an initial clinical assessment. With these criteria for inclusion, the study included only current users. The lower limit of four months was chosen to include more than one AAS cycle, thus indicating regular use.

### Selection of subjects

The patients were primarily selected in order to exemplify variation in the possible combinations of drug use. We also wanted to include both men and women in order to illustrate gender-related differences. Another important criterion for selection was that the narratives should be detailed and the content richly described. Although the cutoff point for AAS use was four months, the selected subjects had a regular use of AAS from nine months to sixteen years.

### Interviews

Prior to the interview, each patient was asked to write down a description of how their drug use had developed over the years, including the names of the drugs they used and when they began using them. The semi-open face-to-face interviews [30] were conducted by one of the authors (KS), who has many years of experience in training and instructing at gyms and who consequently has a good understanding of the environment with which AAS are usually associated.

The interview was carried out as a conversation in which the patient was given considerable freedom on how to tell their life story [31]. Open-ended questions were used in the interviews and they were posed in such a way that the patient was encouraged to relate their experiences as fully and freely as possible. The order in which the questions were posed varied and the interviewer tried to adhere as much as possible to the patient's chosen method of narration. However, at the end of the interview, the inclusion of all the areas of interest was checked. The following areas were covered in the interview:

- childhood, family situation, school experiences

- reason for and situation in which AAS usage was begun

- progress of drug usage

- abuse of other substances

- times at which various substances were first used

- experience of side-effects

- reason for seeking care

The interviews took between one and three hours per patient. The narratives were written down during the interview [32] and the material was then compiled into a personal, chronologically arranged narrative for each informant [33]. The patients were then given the opportunity to read and comment on these texts and to assess whether they seemed reasonable and whether they wished to remove any part. All information that might enable identification of the person was removed in order to guarantee anonymity, and all names used in this article are therefore fictional. The life stories were formulated as closely to the language in which the story was told as possible and in such a way that the informants recognized them as being accurate and in accordance with their experience.

### Ethical approval

The procedures used in this study have been approved by the regional ethical vetting board (No.: 538/99) in accordance with the Swedish law concerning approval of medical research and the patients have given their informed consent.

## Results

### Case I – John, 25

#### The development of early combined drug use starting with AAS

John had a difficult childhood. He felt that he did not receive any love from his mother since she did not bother much about him. He was also subjected to sexual abuse by a relative. He was slender during his teens and bullied by his classmates. For this reason he began training at a gym at the age of 16. His goal was to increase both his strength and his body mass. After four years of training, at the age of 20, one of his gym mates advised him to start taking AAS to enhance the effects of the training, which he did. He soon noticed considerable effects on his training and also enhanced emotional well-being.

After using AAS for some time, he took the advice of some more experienced gym mates and began taking anti-estrogens in order to prevent gynecomastia. He also started using ephedrine, other bronchodilators and dietary supplements that contained ephedrine in order to make him more energetic and to enable him to train harder. He felt both psychologically and physically well when taking ephedrine and started using this even when not using AAS. He also began taking testosterone releasers to speed up his own hormone production. The tough training regimen he now followed led to pain in his muscle insertions and ligaments, which prompted him to begin also taking analgesics. John trained regularly and heavily, sometimes several times a day.

His social interactions became increasingly limited to other AAS users and his knowledge about the drugs and their effects grew rapidly. As a child he was very shy, particularly in relation to girls. He had no contact with girls but instead developed sexual fantasies that occupied a great deal of his time and that has continued into adulthood. When he was twenty-four years of age, he met a woman at the gym and they embarked on a relationship, which was a new experience for him.

In this period of life, the most important thing for him was training at the gym and his life became increasingly focused upon medication, diet and training. In order to train even more, he began using amphetamines; he felt that this helped him to keep alert during training. His experience was that amphetamines allowed him to train even harder. Amphetamines made him feel good mentally but also led to difficulties in relaxing after training. He therefore began taking hashish and benzodiazepines to help him wind down and sleep better. He was now using amphetamines more frequently because he found them to be wonderful for recreational use. John had previously drunk alcohol sparsely but now began using alcohol more frequently to help him sleep and as recreation at the weekends.

Altogether, John was taking fourteen human and veterinary AAS products during a period of five years (oral: oxymetholone, stanozolol, methandrostenolone and methenolone acetate; injected: trenbolone acetate, testosterone blends, boldenone, nandrolone esters, methenolone and stanozolol). Throughout the training period he ate or drank dietary supplements (e.g. protein and various products containing protein, creatine and ephedra) with the purpose of enhancing the effects of training.

Initially, John felt the positive effects from his AAS use far outweighed the negative. He describes increased self-confidence, improved libido and affirmation from both men and women in his surroundings. However, despite using various medications to counteract psychological and physical problems, he experienced more and more negative effects. He experienced testicle shrinkage, skin lesions and potency problems. He also began to experience hallucinations, depression, mood swings, aggressiveness and feelings of persecution. His sexual fantasies also became more marked.

By the age of 25, after five years of AAS abuse, he was tested for AAS use at the gym at which he trained. His regimen at this time included nandrolone decanoate and amphetamine. When the tests proved positive, he was barred from the gym. John cites this as the stimulus for his increasing use of amphetamine and alcohol, although he discontinued both training and AAS. His use of other drugs of abuse and alcohol worsened, with associated severe social problems. His company ran out of business, his girlfriend left him, he failed to pay his rent and he became destitute. It was in this situation that he sought help from the addiction clinic, mainly because of his psychological problems.

### Case 2 – Joe, 37

#### The development of late combined drug use starting with AAS

Joe grew up with his biological parents. He was and remains very close to his mother but was often beaten by his father, with whom he had a very remote relationship. Joe describes his upbringing as very strict because of his father's principles. At school he was often afraid and teased for being small. However, he completed his schooling with top grades. In his early teens, he was prescribed analgesics for frequent headaches and he has continued to take them ever since.

At 16 years of age, he started training at the bench press at a gym and, at the same age, he began drinking alcohol. He drank a fair amount of alcohol in his late teens but, because he felt it impaired his training, he decided to completely quit alcohol in his early twenties. When he was 21 years of age, he felt he had reached a plateau in his training. A friend told him to try AAS to enhance his training. His first course consisted of oral AAS (stanozolol, oxymetholone and testosterone undecanoate) and the associated rapid improvement in strength he experienced prompted him to continue using AAS.

Joe combined AAS with ephedrine and other ephedra preparations to perk himself up. He also took dietary supplements such as protein powder and other protein supplements, creatine, nutritional replacements and multivitamins. He learned from other AAS users at the gym that he could also add anti-estrogens and testosterone releasers in order to counteract the unwanted effects of AAS. He has had to use a number of medications to counteract what he believed to be side effects from his AAS use and hard gym training, including analgesics for pain from over-training, benzodiazepines for insomnia, and analgesics for headaches and pain in muscles and joints.

He combined a painkiller containing codeine with water in a plastic bottle from which he drank continually while at the gym so that he could train harder and longer.

He also took muscle relaxants immediately after a training session. Altogether, he used thirteen different AAS medications during 16 years of AAS abuse (oral: fluoxymesterone, methandrostenolone, methenolone acetate, oxymetolone, stanozolol and testosterone undecanoate; injected: nandrolone esters, stanozolol, several testosterone injections, testosterone blends and trenbolone acetate).

He felt that his self-confidence was much improved when using AAS and he described experiencing better control of his feelings so that he never felt afraid when he was in a confrontational situation. He became stronger and gained weight and felt that his healing capacity was improved. With time, however, the negative effects increased in number and severity. He had previously found it easy to mix with girls. Now he became markedly jealous, had violent mood swings, outbreaks of aggression and frequent depression. He also describes an emotional numbness in relation to others. The physical problems included wear and tear of his joints, testicular atrophy, gynecomastia, acne, blood in his urine, kidney pain and infected skin lesions. The cost of supporting his drug use also continued to rise, leading to criminal behaviour.

At the age of 30, he began using other drugs of abuse, including amphetamines and cocaine. Initially, he took these drugs to increase his ability to train but later he also started taking them at parties for recreational purposes. His other drugs of abuse increased rapidly in number and he began using hashish as well. He sought treatment because of his narcotics use and for the troubling physical and psychological problems he believed were derived from AAS.

### Case 3 – Sune, 24

#### The early development of a complex usage of hormone preparations

Sune had good contact with his father and siblings during his childhood, but contact with his mother was not as good and he describes her as having alcohol and psychological problems. Sune had many friends at school and has maintained contact with several of them later in life. He was never bullied or the victim of any kind of violence. The only problem he recalls from childhood was that he became aggressive rather easily.

He began training at a gym with some friends at about 15 years of age and, when he was 16, he and some friends became curious about whether AAS would give supplementary effects. Even before he began trying AAS, Joe had started using various dietary supplements such as protein and creatine. The first AAS he bought was a testosterone product that was to be injected into the buttock. He experienced clear positive results from this, predominantly as an increase in weight and strength. He noted, however, that he became more irritable. He soon began using AAS more steadily. He used oral AAS (methandrostenolone, stanozolol), injectible varieties (nandrolone esters, different testosterone blends and trenbolone cyclohexylmethylcarbonate) and a fluid form of AAS (unknown name) that could be administered as drops under the tongue.

He later combined AAS with growth hormone and insulin. He took these hormones hoping that this combination would produce even quicker muscle growth. He also started using an anti-estrogen so as to reduce the risk of gynecomastia and testosterone releasers to enhance his own hormone production. Other substances that were added later were ephedrine, prohormones, anticatabolics and testosterone boosters. He was able to train harder and more frequently while using AAS; however, pain in his muscle insertions and joints soon developed. He therefore started taking analgesics in order to be able to train despite the pain.

When he started using hormones, he also started taking protein and creatine supplements and various plant steroid compounds. After four years of abuse and 10 different human steroid products, when he was about 20 years old, his regimen consisted of AAS drugs (nandrolone ester, methandrostenolone) in combination with insulin, testosterone releasers and ephedrine.

Sune experienced mainly positive effects from AAS, particularly in the beginning. He mentioned increased strength and weight gain above all but also a feeling of attractiveness to girls. His sexual drive was considerably increased after the debut of AAS. Sune was, however, at that time also troubled by hair growth on his back, skin lesions between his shoulder and chest musculature, acne, potency problems, testicular atrophy and a cough that bothered him particularly after taking testosterone preparations. He had also suffered serious psychological problems such as pathological jealousy, mood swings, depression and aggressiveness. Several times he had become so angry that he smashed up the furniture at his parents' house. He also attempted to commit suicide. Sune also sometimes had memory problems and his fixation with his body was greatly increased. His parents contacted the addiction clinic because they felt their son's personality had undergone such a radical change.

### Case 4 – Bill, 25

#### The development of body fixation and a complex usage of hormone preparations

Bill describes his childhood in glowing terms. He had good contact with his parents and a younger sister. He was very active in sports such as football, ice hockey, boxing and tae kwando while he was growing up. After leaving home at the age of 15, he stopped these sports and took up training at a gym instead. His training became so intense and time-consuming that his schoolwork began to suffer. He completed his schooling with poor grades.

After a few years of training, he became increasingly focused on competing in the field of bodybuilding. Bill had read about AAS and, while he was thinking about starting to compete, he felt he needed to begin taking AAS so as to increase his body size, since he believed everyone in elite level bodybuilding was using AAS. He was 20 years old when he began taking AAS. The first course consisted of oral methandrostolone and injections of testosterone blends. He described the positive effects as including increased body bulk and strength as well as a powerful "pump" feeling, particularly in his biceps, when he was training. He described the feeling when the blood pumped into a specific muscle as almost orgasmic. He also described increased libido and significantly enhanced self-confidence that meant he "felt like a king in the town". He sought out fights because it gave him a "good feeling" when others were afraid of him. Bill soon became preoccupied with his AAS use and began reading more about preparations, training and AAS. He found advice that prompted him to start using testosterone releasers for speeding up his own hormone production.

He had previously begun using dietary supplements such as protein and creatine and now he added other compounds. His strength and body bulk increased but he wanted to become even bigger. He now began using a combination of AAS and other hormone preparations such as growth hormone, insulin and IGF-1 (Insulin growth factor 1).

During the four years of AAS use, he used a total of nine different human and veterinary AAS preparations: oral AAS consisting of methandrostenolone, stanozolol and testosterone undecanoate and injectible AAS in the form of boldenone, nandrolone ester, various testosterone blends and trenbolone acetate. The whole time that Bill was training, he used stimulants such as ephedrine and sometimes bronchodilators to reduce fat and fluid in the muscle tissues. He has also tested other drugs of abuse such as amphetamines and hashish at parties and has used alcohol occasionally.

The second to last course he took, which included nandrolone esters, testosterone blends, growth hormone, insulin, and testosterone releasers, resulted in such a drastic drop in blood sugar level that he was hospitalized. The first time he sought help at the addiction clinic he described himself as severely depressed. The reason he gave was that he had gone from weighing 128 kg to, as he said, "only 124 kg". He said he could not imagine going back to his old gym where everyone would see how "small he had become". In order to gain weight quickly he therefore began his last course of AAS. During this course, he had such drastic physical and psychological problems that he decided to completely stop using AAS.

Altogether, he suffered a range of physical problems such as breast development, acne, skin lesions, testicular atrophy, reduced libido and fatigue. Psychologically, he felt depressed, with mood swings, increased aggressiveness, panic attacks and pronounced body fixation. Sometimes, under the influence of AAS, he would wander around the streets of his hometown looking for fights because he felt himself to be invincible. While using AAS, he developed a criminal career and was sentenced several times for various acts of violent crime.

### Case 5 – Irene, 26

#### The development of the use of enhancing drugs and an extreme body fixation

Irene describes her childhood as very problematic. She felt she was pushed aside as a child because her brothers always came first. She also experienced sexual harassment by a close relative and was bullied in school. In her early teens, she became increasingly fixated with her body, constantly asking others what they thought of it. Irene completed her schooling with poor grades.

As a teenager she was very active in several sports and at the age of 17 she began training at a gym as a complement to her handball training. She felt good and she believed that her body became more beautiful thanks to the tough gym training. She soon quit her other sports and decided to begin gym training to compete in bodybuilding. She was then convinced that a prerequisite for success in this sport was the use of AAS, and this led to her AAS debut at the age of 20. The first course, which lasted three months, consisted of stanozolol injections. She felt that, once she had begun using AAS, her body fixation intensified. During the second course, she took not only AAS injections but also growth hormone (hGH). Her psychological problems, with mood swings, anxiety and irritation, worsened.

She soon began experiencing more physical problems, such as clitoris enlargement, hair loss and yellowing of her skin. In the six years during which she used hormones, she used four different types of AAS (oral: methandrostenolone and stanozolol; injected: methenolone enanthate and stanozolol). She also took several courses that included hGH. Early on in her AAS use, she also used ephedrine and other preparations to reduce subcutaneous fat and fluid in the muscles (e.g. bronchodilators and a drug which contained a combination of ephedrine, caffeine and aspirin).

Irene trained far harder after starting to take hormones. Sometimes she would train several times a day, which led to pains in her muscle insertions. She began taking analgesics in order to continue training despite the pain. She became much stronger after starting to use AAS, and she then preferred to train with men. Because she was afraid of building up fat or retaining fluid, she has only occasionally used dietary supplements over the years and, when she has done so, has primarily taken protein supplements. She tested creatine but stopped because of weight gain. Irene was just over 26 when she stopped using AAS after a final course consisting of oral methenolone acetate, an ephedra preparation, ephedrine tablets and bronchodilators.

Irene describes the positive effects she experienced with AAS as increased muscle bulk, a harder body and a psychological boost including improved self-confidence. However, she also notes psychological problems that at times were considerable, such as jealousy, extreme body fixation, powerful mood swings, aggressiveness and recurrent depression including suicidal fantasies.

She has tried various anti-depressant medications, all of which she discontinued because she felt they made her retain fluid. She has also undergone breast enhancement surgery and her voice became deep. Irene describes how she became very popular among men and she had an increased sex drive leading to unfaithfulness. She has lived in several partnerships but all of them broke up because of her body fixation and her extreme jealousy, which often led to maltreatment of partners. Irene sought treatment at the addiction clinic for her psychological problems, particularly for her fixation with her body. Before she came to the clinic she had met several doctors but had not found them helpful since they knew so little about AAS.

### Case 6 – Sonja, 22

#### The development of oscillating drugs of abuse and AAS use

Sonja grew up with her biological mother and an older sister. Their parents divorced when the girls were very young and their mother refused to allow them to meet their father. Sonja describes her upbringing as slack. Their mother was described by Sonja as selfish and the girls were allowed to do as they pleased. Her schooling was rife with problems and she quit school at the age of fourteen. She found it difficult to concentrate in class and often fought with her teachers and with other students. Sonja became more interested in sports after leaving school and involved herself in several, including running and swimming.

She travelled overseas to work at the age of 17 and remained there for one and a half years. After this, she returned home and met a man who was using other drugs of abuse. Her contact with him was her gateway into drug use, and he introduced her to amphetamine. Sonja then stopped all her sports and instead developed quite a severe problem with drug use, including heroin and other opiates, amphetamines and analgesics. Later, she also included hashish and cocaine.

At the age of 21, she met a man who was using AAS. Under his influence, she also started to train at the gym. Since she wanted to have a larger and stronger body, she began using AAS (oral methandrostenolone), and she discontinued her use of drugs of abuse. Sonja's self-confidence improved and she felt generally good taking AAS, which led her to continue with testosterone undecanoate and stanozolol. While taking this course, she noticed that her skin became greasier, her hair looked unwashed, and she had more acne, mood swings and outbreaks of aggression. She was now training seven days a week at the gym, sometimes twice a day. She began to take dietary supplements such as protein, creatine, vitamins and sometimes CLA (Conjugated Linoleic Acid) to keep her weight under control.

Her aggressiveness worsened after starting AAS use and she increasingly frequently got into fights in order to find release for these feelings. She began walking around town looking for someone to fight with because fighting gave her a sense of satisfaction. She felt she was truly alive on these occasions. She bought herself a dog, which she also beat when it did not behave. During her nine months of AAS use, she used oral methandienone, testosterone undecanoate and stanozolol. She also took ephedrine and clenbuterol before training. After a drug-free period she once again began taking other drugs of abuse, although in smaller quantities than before.

She finally stopped taking AAS after encountering problems such as pain and acne but above all because of her aggression and suicide attempts. She was also arrested several times for her involvement in fights. She had previously been sentenced for theft but her criminality increased markedly after she began using AAS. She was then sentenced for drugs of abuse and doping offences. She sought treatment for her drug problem, which had increased to include ecstasy, amphetamines, buprenorfin and benzodiazepines.

## Discussion

As far as we are aware, this study is the first in which patients from an addiction clinic describe the development of their multiple drug use including doping agents (hormone preparations sometimes in combination with other drugs) from a subjective perspective. A primary finding from the patients' narratives is that the use of AAS can develop under widely varying conditions in terms of social background, timing of initiation, development of multiple drug use, and the associated physical and psychological problems. Despite these significant variations, certain common features in the patients' stories are discernible.

Most of the patients in this study describe childhoods with many problems, including physical or psychological abuse. Their problems extended into their time at school and affected both their social and academic achievements. In an earlier study [28], we found social problems to be highly overrepresented among AAS users compared with gym users who were not taking drugs. Negative experiences of school-mates have also been revealed in other studies [34]. It is important to remember, however, that some AAS users describe positive childhoods, which means that there is no straightforward relationship between upbringing and abuse of AAS [28].

All of the patients in this study began using AAS in association with gym training. Most of them were in their late teens, which tallies with earlier reports [6]. The use of AAS continued for between nine months and 16 years. This variation in the duration of AAS use reflects the variations found in clinical addiction treatment practice. For four of these patients, AAS was the first drug they had ever used, while one of them had used alcohol as a first drug and another had used other drugs of abuse (predominantly amphetamines). The only gender-related differences we noticed were that the women used fewer AAS drugs than the men.

In this study, we found that the participants started gym training with the addition of dietary supplements and were later advised to add AAS and other hormones to enhance the effects of training. To prevent AAS-related problems and to enhance the AAS effects, they added various pharmaceuticals, such as ephedrine, testosterone releasers and anti-estrogens, and also alcohol. Some of them also later added other drugs of abuse, such as amphetamine, to further enhance the effects on their training.

A common reason for taking AAS seems to be the experience of reaching a plateau in training effects, leading them to seek possibilities for enhancement. As noted in an earlier study [35], others started AAS to increase body size and muscle strength. Two patients in our study who began using AAS because they wished to compete in bodybuilding believed that AAS use was essential for success in this field. It is of interest that neither of these two patients mixed the hormones with other drugs of abuse.

For two other informants, use of AAS was soon associated with use of other hormone preparations, different drugs of abuse, medications, alcohol, and dietary supplements. This was, however, not the case for the two who wished to compete. The reasons given for the increasing numbers of preparations were to increase the effects of training and the effects of the AAS or to reduce what were believed to be side effects of AAS. In a case description by Wilson-Fearon and Parrot, a male bodybuilder described how he used a cocktail of drugs before competing [36] and Pope and Kanayama [24] describe a case of an AAS user starting to use opioids after getting "pain in his 'delts' from military presses".

Several of the patients spoke of a great interest in learning more about AAS and other hormone preparations. They readily talked about the underground literature (books, magazines) and web sites where detailed descriptions could be found of which preparations and drugs can and, according to some authors, should be taken with AAS. The fact that information is sought through these media has also been noted in a previous study [37].

The knowledge held by many patients about combining various preparations has clearly become extensive after taking the drugs for some time. This indicates that they felt that their careers were dependent on their considerable knowledge about which drugs can be taken in combination with AAS. In a study by Grogan et al., this was reflected in the comment "I know more than my doctor", particularly when it came to knowledge of the positive and negative effects of AAS [26].

The subjective experience of AAS varied in type and severity but was pronounced and associated with considerable medical and/or psychological problems in all patients. The most commonly reported physical problems were changes in sexual potency (increased and/or decreased libido), skin lesions, testicular atrophy, acne and gynecomastia. Among the commonly reported psychological side effects were mood swings, aggressiveness, depression, jealousy and increased fixation with body image. These problems are commonly reported by AAS users, for example on the Swedish anti-doping hot-line [7].

Aggressiveness affected four of the patients (two men and two women) and prompted three of them to actively seek out fights. The fourth patient, who already had problems with aggressiveness before using AAS, was the only one who reported aggressive breakthroughs as "roid rage". In a study by Wilson-Fearon, a competitive body builder described how he had to quit work as a security guard several weeks before competing because of problems in controlling his aggressiveness [36].

Pathologically extreme jealousy was a major problem for four of the patients, causing severe disruptions in their relationships. Some of the other problems the patients reported included pain, hair loss or hair growth, clitoris enlargement, unfaithfulness, suicide attempts or suicidal thoughts, and emotional numbness. This emotional numbness was, however, seen as desirable by some informants since it facilitated fighting.

An important finding in this study is that most of the patients describe their early experiences of AAS as definitely positive, perhaps even as the best time of their lives. Olrich and Ewing showed that three common positive effects from AAS use were improvement of one's social status, positive peer recognition and improved vocational performance (increases in work effectiveness, alertness at work and confidence at work) [27]. The most common positive effects described by the patients in this study were increases in strength, body bulk and self-confidence. However, the patients also said that, as their AAS use continued, the negative experiences began to outweigh the positive experiences and that this development was a necessary prerequisite for seeking treatment.

The results of this study should be viewed in light of the fact that the sample is small and specifically selected to represent the wide variations in the development of AAS abuse that we have noted in our clinical work. It should be noted that, consequently, quantitative conclusions couldn't be drawn from this study. In an earlier study [28] we noted, however, that most AAS users at an addiction clinic had social problems from their childhoods with respect to both family and schooling. In another study [29], we also showed that AAS use is often associated with use of other drugs of abuse, pharmaceuticals and alcohol.

## Conclusion

This study shows the wide variation in patterns of development of multiple drug abuse in users of AAS. Earlier studies have demonstrated that multiple drug use is common. This study adds information on how this development can occur along different paths and for different reasons, and indicates that AAS can be a gateway to the use of other drugs of abuse. The stories told by the users provide information about AAS use from a subjective perspective, which can be important when designing treatment programmes that are adapted to this special group of patients. By listening to the patients, we can learn about what can trigger an interest in AAS, how multiple drugs can be added and what positive and negative effects can be experienced. This knowledge could help counteract the low levels of trust that AAS users often show towards health care providers.

Our objective was not to make broad generalizations but rather to show the wide variation in the patterns of development of preparation use and effects on users of AAS. We contend that care providers should see their task as two-fold. Firstly, it is important as a care provider to possess a high level of general knowledge about AAS use and the possibility of concomitant drug use in order to instill confidence in the patient at the outset of treatment. Secondly, it is important that the care provider avoids stereotypical notions of how abuse usually develops since it can take a variety of forms and have a variety of outcomes. Good general knowledge and an interest in the individual patient's particular life experience are two equally important factors in working with AAS users.

The information from this study may also be useful for policy planning. It is important that the designers of abuse prevention programmes understand the reasons for starting using AAS in order to develop a fact-based message for target groups. This information may also be important in the development of policies concerning detection of abuse and the development of assistance programmes, since AAS users often experience a range of highly desirable effects from the drugs and only seek treatment as an alternative when the negative effects outweigh the positive effects.

## Competing interests

The authors declare that they have no competing interests.

## Authors' contributions

KS conceived the idea for the study, participated in its design, carried out all interviews, took part in the analysis of results and drafted the manuscript. FN was active in the analysis of results and helped to draft the manuscript. IE was responsible for the design of the study and helped to draft the manuscript. All three authors have read and approved the final manuscript.
